# Draft genome sequences of halotolerant *Halomonas* spp. SpR1 and SpR8, potential plant growth-promoting bacteria associated with *Salicornia* rhizosphere in a hydrothermal lagoon ecosystem of the Altiplano, Northern Chile

**DOI:** 10.1128/MRA.00822-23

**Published:** 2023-12-04

**Authors:** Inaudis Álvarez-Hubert, Roberto E. Durán, Paulina Vega-Celedón, Ingrid-Nicole Vásconez, Constanza C. Macaya, Michael Seeger

**Affiliations:** 1 Laboratorio de Microbiología Molecular y Biotecnología Ambiental, Departamento de Química & Centro de Biotecnología Daniel Alkalay Lowitt, Universidad Técnica Federico Santa María, Valparaíso, Chile; Montana State University, Bozeman, Montana, USA

**Keywords:** *Halomonas*, Altiplano, Chile, genome analysis, *Salicornia*, hydrothermal lagoon

## Abstract

Halotolerant *Halomonas* spp. SpR1 and SpR8 are potential plant growth-promoting bacteria (PGPB) isolated from *Salicornia* rhizosphere in a Chilean Altiplano hydrothermal lagoon. We report draft genomes of *Halomonas* sp. SpR1 (5.17Mb) and *Halomonas* sp. SpR8 (4.47 Mb). Both represent potentially novel independent species closely related to *Halomonas boliviensis* DSM 15516^T^.

## ANNOUNCEMENT

Soil salinity stress limits plant growth and reproduction. The application of plant growth-promoting bacteria (PGPB) under stress conditions constitutes an emerging strategy for enhancing agricultural production (1, 2). Genome-guided analyses are useful for the identification of halotolerant PGPB and their molecular mechanisms that contribute to alleviating crop stress in saline soils (1, 3).

*Halomonas* strains inhabit a range of environments, including plants (4). We report the draft genome sequences of two strains isolated from the rhizoshere of *Salicornia* sp. (Salicornioideae), a succulent halophyte plant (5) collected on the shore of Laguna Roja, Amuyo, Parinacota Region, located in the Northern Chilean Altiplano at 3,800 m.a.s.l. (19°3′29.876″S; 69°15′10.455″W). The rhizosphere soil was collected and kept at 4°C until processing. For bacterial isolation, 4 g of soil was mixed with saline solution (NaCl 0.85% wv^−1^). Next, 100 µL of serial dilutions of the suspension was spread on nutrient agar (NA) supplemented with NaCl 7% wv^−1^ (pH 7) and cycloheximide (100 µg mL^−1^) and incubated at 25°C for 1 week (2). Morphologically distinct individual colonies were plated on NA until pure strains were obtained. Both strains were selected due to their salinity tolerance (0% to 14% NaCl wv^−1^).

Genomic DNA was extracted from one colony on an NA plate using a modified Marmur technique (6). DNA was sequenced using Illumina MiSeq paired-end technology (2 × 250 bp30X) in MicrobesNG (Birmingham, UK). Libraries with a median insert size of 504 bp (SpR1) or 487 bp (SpR8) were generated with a Nextera XT library preparation kit (Illumina) following the manufacturer’s guidelines. A total of 2,238,744 (SpR1) and 1,271,056 reads (SpR8) were recovered. Whole genome sequencing reads were adapter-trimmed with Trimmomatic 0.30 using a sliding window quality cutoff of Q15 (7). *De novo* assemblies were performed using SPAdes 3.15.4 (8).

The draft genome of *Halomonas* sp. SpR1 comprises 96 contigs with a size of 5,168,778 bp (G+C content: 54.94%; average coverage: 97.6×; N_50_: 120,216 kb). In *Halomonas* sp. SpR8, the draft genome comprises 31 contigs with a size of 4,470,281 bp (G+C content: 54.68%; average coverage: 61.9×; N_50_: 351,539 kb). The N_50_ value was calculated using PRINSEQ 0.20.4 (9). The genome annotations were performed using the Prokaryotic Genome Annotation Pipeline (10), identifying 4,784 and 4,080 coding sequences for SpR1 and SpR8, respectively. Default parameters were used for all software.

**Fig 1:**
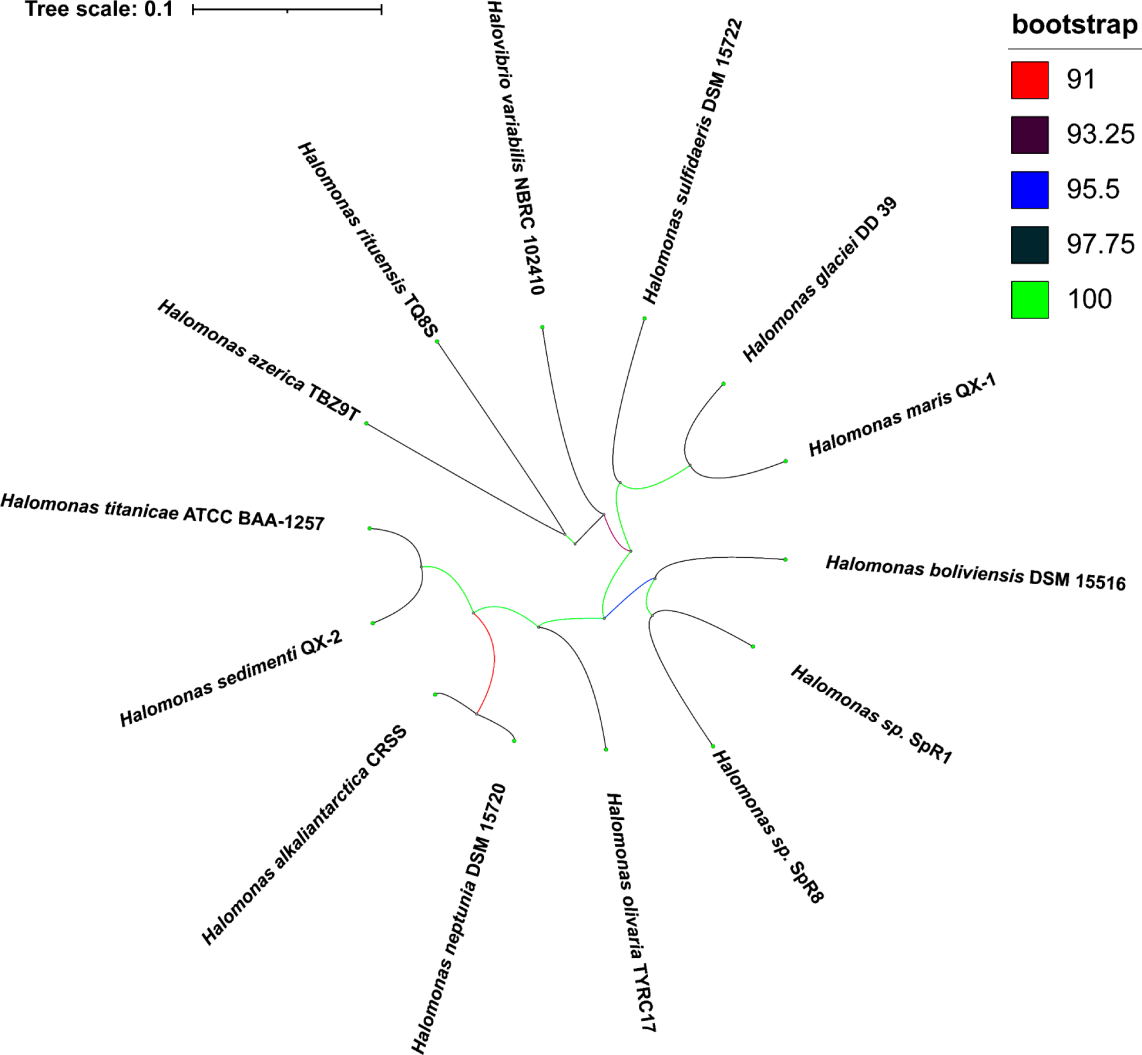
The tree of *Halomonas* strains SpR1 and SpR8 generated from Genome Blast Distance Phylogeny (GBDP) distances obtained from genomic sequences with FastMe 2.1.6.1 (13). The branch lengths are equal to the GBDP distance formula, d5. Branch colors represent GBDP pseudo-bootstrap support values greater than 60% from 100 replications, with an average branch support of 98.3%. The tree was rooted at the midpoint (14) and rendered using iTOL 6.7 (15).

The draft genome sequences were submitted to the JSpecies WebServer 4.0.2 and the Type (Strain) Genome Server for a whole genome-based taxonomic analysis (11, 12). *Halomonas* spp. SpR1 and SpR8 grouped in the same clade (digital DNA-DNA hybridization, dDDH-d4, 34.3%), with an average nucleotide identity (ANIb) value of 87.17%, each representing an independent species closely related to *Halomonas boliviensis* DSM 15156^T^ (accession number GCA_000236035) (Fig. 1). The dDDH values for each strain in comparison to the closest type strain species, *H. boliviensis* DSM 15156^T^, supported their identification as two potentially novel species within the genus *Halomonas* [*Halomonas* sp. SpR1 (dDDH-d4 29.9%, ANIb 87.36%); *Halomonas* sp. SpR8 (dDDH-d4 34.2%, ANIb 85.19%].

*Halomonas* spp. strains SpR1 and SpR8 potentially synthesize betaine, ectoine, and 5-hydroxyectoine. AntiSmash 7.0 software revealed non-ribosomal peptide biosynthetic gene clusters that encode enzymes for the synthesis of potashchelin-type metallophore (identity: SpR1 84%, SpR8 75%) (16, 17).

## Data Availability

*Halomonas* spp. SpR1 and SpR8 draft genome sequences were deposited in GenBank under the accession numbers JAUAMB000000000 and JAUAMC000000000, respectively. The raw sequencing data for strains LRA-SpR1 and LRA-SpR8 are available in the SRA under the accession numbers SRR24780816 and SRR24780815, respectively.
